# Validation of a Stability-Indicating Hydrophilic Interaction Liquid Chromatographic Method for the Quantitative Determination of Vitamin K_3_ (Menadione Sodium Bisulfite) in Injectable Solution Formulation

**DOI:** 10.3797/scipharm.1303-05

**Published:** 2013-05-09

**Authors:** Mashhour M. Ghanem, Saleh A. Abu-Lafi, Hussein O. Hallak

**Affiliations:** 1 Pharmacare Pharmaceutical Company, P.O. Box 677, Ramallah, Palestine.; 2 Faculty of Pharmacy, Al-Quds University, P.O. Box 20002, Abu-Dies, Palestine.

**Keywords:** Menadione sodium bisulfite, Menadione sodium bisulfite injectable solution, Validation, Stability indicating method, Liquid chromatography, Vitamin K

## Abstract

A simple, specific, accurate, and stability-indicating method was developed and validated for the quantitative determination of menadione sodium bisulfite in the injectable solution formulation. The method is based on zwitterionic hydrophilic interaction liquid chromatography (ZIC-HILIC) coupled with a photodiode array detector. The desired separation was achieved on the ZIC-HILIC column (250 mm × 4.6 mm, 5 μm) at 25°C temperature. The optimized mobile phase consisted of an isocratic solvent mixture of 200mM ammonium acetate (NH_4_AC) solution and acetonitrile (ACN) (20:80; v/v) pH-adjusted to 5.7 by glacial acetic acid. The mobile phase was fixed at 0.5 ml/min and the analytes were monitored at 261 nm using a photodiode array detector. The effects of the chromatographic conditions on the peak retention, peak USP tailing factor, and column efficiency were systematically optimized. Forced degradation experiments were carried out by exposing menadione sodium bisulfite standard and the injectable solution formulation to thermal, photolytic, oxidative, and acid-base hydrolytic stress conditions. The degradation products were well-resolved from the main peak and the excipients, thus proving that the method is a reliable, stability-indicating tool. The method was validated as per ICH and USP guidelines (USP34/NF29) and found to be adequate for the routine quantitative estimation of menadione sodium bisulfite in commercially available menadione sodium bisulfite injectable solution dosage forms.

## Introduction

**Fig. 1:**
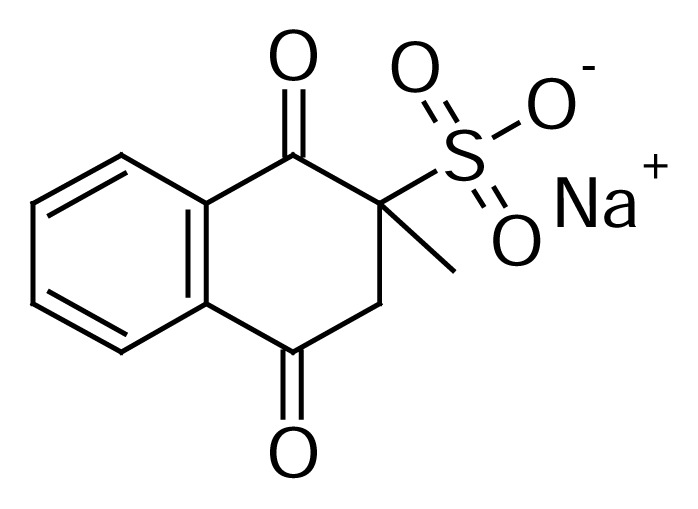
Chemical structure of menadione sodium bisulfite

Menadione sodium bisulfite injectable solution is a veterinary drug which comprises vitamin K_3_ in the form of menadione sodium bisulfite (MSB) as the active ingredient and a mixture of inactive excipients. MSB is a synthetic vitamin K compound that is used for the treatment of prolonged bleeding due to vitamin K deficiency states, which occurs in cattle fed moldy sweet clover containing dicumarol [1]. Figure 1 shows the chemical structure of the MSB active ingredient in MSB injectable solution.

There are few analytical HPLC methods that describe the HPLC analysis of vitamin K_3_ individually. Some of these methods utilized reversed-phase ion-pairing with UV detection [2], dual-electrode amperometry [3], fluorescence [4, 5], and electrochemical detection [6]. Recently, we have successfully developed a novel validated HPLC method for the simultaneous determination of MSB combined with amprolium hydrochloride and sulfaquinoxaline sodium in the powder formulation [7]. The official BP and USP methods for MSB analysis rely on the titration of menadione as an active ingredient [8, 9]. Another USP method analyzed for menadione in the injection formulation [10]. The method requires laborious sample preparation procedures and derivatization with the 2,4-dinitro-phenylhydrazine reagent to increase the MSB extinction coefficient followed by UV absorption spectroscopy measurements [10]. The use of UV-Vis spectrophotometric measurements could not be considered as a stability-indicating procedure since the degradation products may interfere positively or negatively with the absorption measurements. Moreover, the method is time consuming, labor intensive, and utilizes expensive and environmentally hazardous solvents.

MSB injectable solution contains benzyl alcohol preservative which absorbs strongly at the same UV region of MSB. Moreover, the degradation products produced upon exposing MSB to stress conditions also absorb at the same UV region of MSB. Consequently, the use of UV absorption spectroscopy, which is adopted in USP, cannot be used as a selective stability-indicating method for MSB analysis in the injectable formulation.

According to the literature, there is no stability-indicating HPLC method reported yet for the determination of vitamin K_3_ either individually or in combination. Therefore, there is a need to develop a simple, precise, validated, and stability-indicating quality control method that allows for the determination of MSB in the injectable solution formulation.

The MSB active ingredient has a polar and hydrophilic acid character (Figure 1). Therefore, it would elute within the column’s dead volume using a typical reversed-phase mode. In order to enhance MSB retention without derivatization or by adding an ion-pair reagent to the mobile phase, zwitterionic hydrophilic interaction liquid chromatography (ZIC-HILIC) was utilized. HILIC-HPLC is a complimentary method to RP-HPLC and is especially used in situations where the compound retention is poor and very high levels of water are required in the mobile phase for adequate retention. Retention in HILIC is believed to be a combination of hydrophilic interaction, ion-exchange, and some reversed-phase retention. HILIC is a straightforward, versatile, and robust separation technique for the separation of polar and hydrophilic compounds [11]. The HPLC method described herein is based on the zwitterionic hydrophilic interaction liquid chromatography (ZIC-HILIC) coupled with ultraviolet detection. It successfully separates MSB from its degradation products and from benzyl alcohol preservative simultaneously. This HILIC-based HPLC method was validated according to the ICH/USP guidelines (USP34/NF29) [12, 13].

## Results and Discussion

### Method Development and Optimization

Initial method development evaluated different percentages of acetonitrile (ACN) and 0.1 M ammonium acetate (NH_4_AC) buffer adjusted to pH 5.5 as the mobile phase on the Octadecyl Silane C18 chemically bonded (250 mm × 4.6 mm i.d., 5μm particles) (ODS) reversed-phase column. The MSB peak always eluted near to the void peak even when the concentration of ACN was reduced to 2.0%. It was expected that the retention on the ODS column would be difficult for MSB since it is a hydrophilic compound. As an alternative, we separately tried two ion-pair reagents of sodium 1-hexanesulfonate and sodium 1-decanesulfonate on the same column. The mobile phases used were a mixture of 1.0 g of each ion-pair reagent dissolved in 500 ml of water; 12 ml of glacial acetic acid, 2.0 ml of triethylamine, with different percentages of methanol ranging from 50 to 450 ml using 100 ml increments. The main observations were that a broad MSB peak was produced that had a relatively high tailing factor. More importantly, the sensitivity of MSB decreased to about 40% relative to the same concentration analyzed on the same column without an ion-pairing reagent. Since utilizing the ion-pairing mode usually shortens the lifetime of the column as well as being an expensive reagent, this prompted us to try hydrophilic interaction liquid chromatography (HILIC) technology.

HILIC methodology is complementary to the reversed-phase mode with the added benefit of being able to particularly withhold polar compounds that cannot be retained on the reversed-phase column. Consequently, the proposed HPLC method was screened by using HILIC with different chromatographic variations such as ACN content, pH, temperature, and diverse ionic strengths of NH_4_AC buffers. The intention was to develop an HPLC-HILIC method that has the capability to separate MSB from the placebo and degradation products obtained from the stress conditions.

According to the column manufacturer recommendation, a typical mobile phase for HILIC technology includes ACN with an ionic additive such as ammonium acetate to control the mobile phase pH and ion strength. Therefore, the ionic strength of the mobile phase was started at a 50mM NH_4_AC concentration and was increased up to 200mM with 50mM increments at a fixed pH of 5.5. It turns out that the ionic strength of the mobile phase has only a negligible effect on the retention of MSB, but the peak tailing factor slightly improved with increased ionic strength. Next, different mobile phase pH values from 3.7 up to 7.2 with 0.5 increments were evaluated. As the pH increased, the retention time of MSB slightly increased. The effect of acetonitrile strength on the retention and tailing factor of MSB was explored using 200mM NH_4_AC at a pH of 5.7. As the ACN percentage increased from 50% to 90% with 10% increments, the retention time increased. The best peak tailing factor with a reasonable retention time of about 7.2 minutes was obtained at 80% ACN. Different temperatures of 15°C, 20°C, 25°C, 30°C, and 35°C were also evaluated. Results indicate that the temperature at this range does not play a tangible role in the retention or the peak shape of MSB and therefore a temperature of 25°C was chosen during the entire study.

The optimal mobile phase chosen with the HILIC column was an isocratic solvent mixture prepared by mixing 200 mM NH_4_AC solution and acetonitrile (ACN) (20:80; v/v), shaken well and left till the temperature of the mobile phase reached room temperature. Then the pH was adjusted to 5.7 with glacial acetic acid. A wavelength of 261 nm was chosen since MSB was found to have a maximum at this wavelength.

**Fig. 2:**
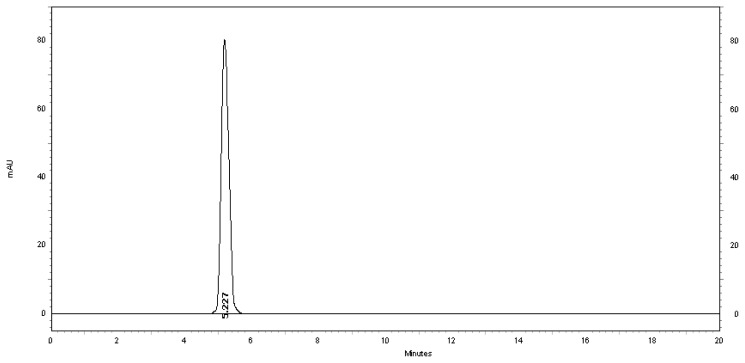
Typical chromatogram of the placebo. The peak at 5.227 minutes is due to benzyl alcohol preservative.

**Fig. 3:**
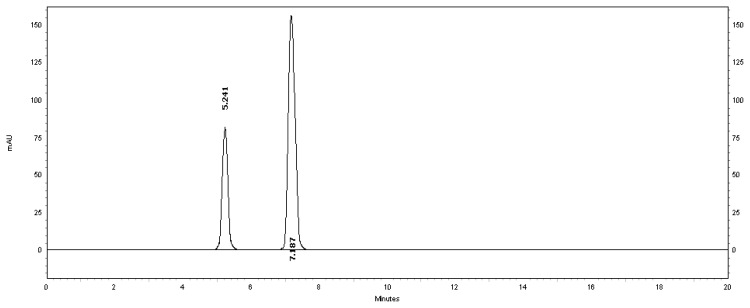
Typical chromatogram of a standard mixture of 30 μg/ml MSB (7.187 minutes) and benzyl alcohol preservative (5.241 minutes).

Figure 2 shows the typical chromatogram of the placebo used at the optimized conditions. Figure 3 also shows a typical HPLC chromatogram of a freshly prepared mixture of MSB and benzyl alcohol preservative using the optimized conditions.

### Method Validation

After the successful optimization of the HPLC-HILIC method, it was validated in accordance to the ICH/USP guidelines [12, 13]. Parameters such as system suitability, specificity (placebo and forced degradation interferences), sensitivity (LOD and LOQ), linearity, range, accuracy (recovery), precision (repeatability and intermediate precision), robustness, and stability-indicating capability were validated.

### System Suitability

The system suitability was determined by injecting six successive replicates of the same standard solution and analyzing the MSB for its peak area, peak USP tailing factor, number of theoretical plates, capacity factor, and resolution between MSB and benzyl alcohol. The system suitability results for a solution of 30 μg/ml MSB revealed the %RSD of less than 1.0% for peak areas. This method meets the accepted requirements as shown in Table 1.

### Specificity (Placebo and Forced Degradation Interference)

Generally, the specificity of a method is its suitability for the analysis of a compound in the presence of potential impurities. Placebo, standard, and sample test solutions were all injected at the same wavelength of 261 nm to assure the specificity of the optimized method. A comparison of the retention times of MSB in sample solution and in the standard solution were exactly the same. Figures 2 and 3 showed that there is no interference at the retention time of MSB due to the placebo. Therefore, the proposed method is suitable for the quantification of the MSB in the MSB injectable solution.

The specificity of the method to MSB was determined in the presence of its stress impurities. It was assessed by performing forced degradation studies on pure standards of the MSB separately to indicate the initial results and on samples of the MSB injectable solution in the presence of its potential degradants. The stress conditions studied were UV-light (254 nm), heat (70°C), acid hydrolysis (0.10 N HCl), base hydrolysis (0.10 N NaOH), and oxidation (1% H_2_O_2_). The stressed sample solutions were analyzed against the freshly prepared standard and sample solutions. The assay and purity check for the stressed standard and sample solutions were calculated as summarized in Table 2.

**Fig. 4:**
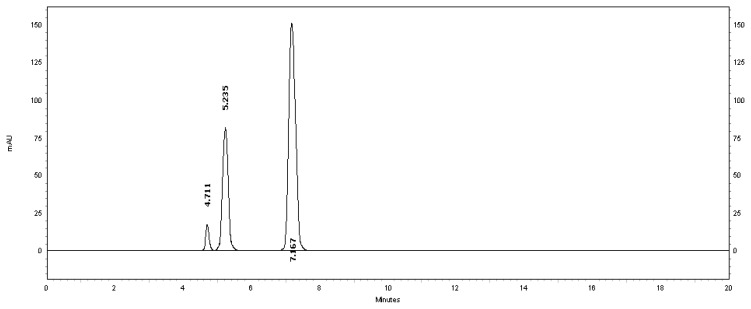
HPLC chromatogram of the MSB injectable solution upon exposure to UV-light for 36 hours, benzyl alcohol (5.235 minutes) and MSB (7.167 minutes). The unknown degraded impurity appeared at 4.711 minutes

**Fig. 5:**
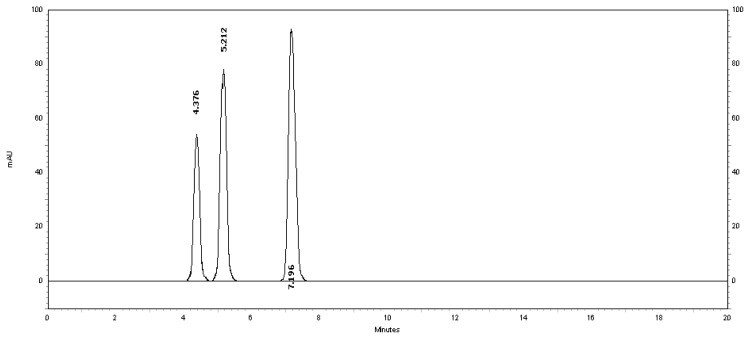
HPLC chromatogram of thermal degradation of the MSB injectable solution upon exposure to heat for 96 hours, benzyl alcohol (5.212 minutes) and MSB (7.196 minutes). The unknown degraded impurity appeared at 4.376 minutes

**Fig. 6:**
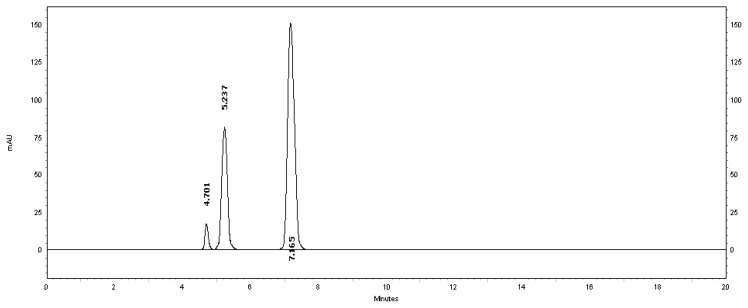
HPLC chromatogram of acidic degradation of the MSB injectable solution after 60 minutes, benzyl alcohol (5.237 minutes) and MSB (7.165 minutes). The unknown degraded impurity appeared at 4.701 minutes.

**Fig. 7:**
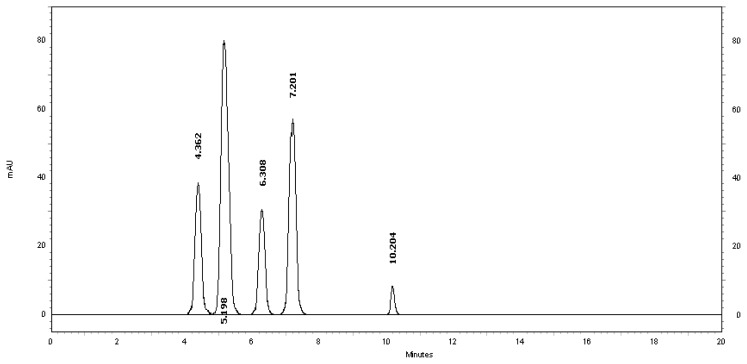
HPLC chromatogram of basic degradation of the MSB injectable solution after 60 minutes, benzyl alcohol (5.198 minutes) and MSB (7.201 minutes). The three unknown degraded impurities appeared at 4.362, 6.308, and 10.204 minutes.

**Fig. 8:**
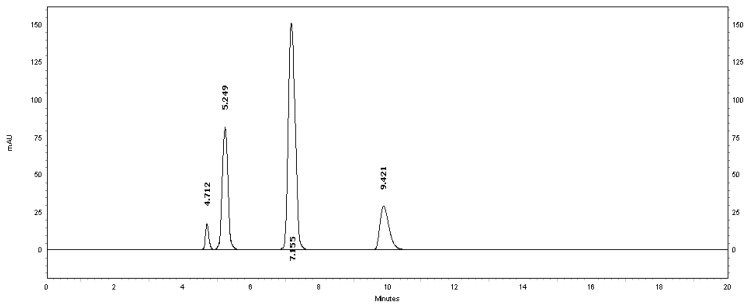
HPLC chromatogram of oxidative degradation of the MSB injectable solution after 24 hours, benzyl alcohol (5.249 minutes) and MSB (7.155 minutes). The unknown degraded impurity appeared at 4.712 minutes. The last eluted peak (9.421 minutes) is due to H_2_O_2_.

Table 2 revealed that the alkaline and thermal stress results showed extensive degradation in comparison to the other stress conditions. The peak purity index for MSB was found to be no less than 0.9986, a higher value than the accepted limit (0.990). Therefore, there was no interference between the MSB peak and any other stress impurity peaks in the chromatogram. Almost the same pattern of degradation was obtained for MSB in the MSB injectable solution samples. Figures 4–8 show the chromatographic profiles of the MSB and the degradation products after exposing the MSB injectable solution to different stress conditions as in Table 2.

### Sensitivity

The sensitivity of the method was explored via measuring the limit of detection (LOD) and the limit of quantitation (LOQ) for MSB at a signal-to-noise ratio of 3 and 10, respectively. It has been achieved by injecting a series of diluted solutions with known concentrations. The LOD was found to be 0.017 μg/ml. The LOQ was found to be 0.057 μg/ml with an RSD of 2.8% (accepted value is less than 10%).

### Linearity and Range

Different amounts of MSB in the range of 50% to 150% of the labeled amount (five concentration levels and three replicates each) were spiked to the MSB injectable solution matrix (placebo).

The linearity in the range of 15–45 μg/ml for MSB was investigated. The regression line demonstrated linearity in the tested range. The regression analysis confirmed that the deviation of the y-intercept from zero is not significant; and the regression line was linear with *R^2^* of 0.9996 (Table 3).

### Accuracy (Recovery)

Accuracy was determined by the recovery study of known amounts of the MSB standard added to a placebo matrix for the injectable dosage form. Different concentrations of the MSB were added to the placebo matrix and the recovery was measured. The data obtained for the evaluation of linearity were used. The accuracy as reflected from the recovery data and the statistical evaluation for the assay of the MSB is listed in Table 4. The average recovery data of MSB showed results between 98.3% and 99.8% with % RSD of less than 1.2%, which are within the acceptable limit of (98.0 to 102.0% recovery and %RSD of not more than 2.0% as set according to the Palestinian Ministry of Health Registration Department criteria).

### Precision

#### Repeatability

One laboratory analyst carried out the assay of MSB on six determinations of the homogeneous sample of the MSB injectable solution at the 100% level of the test concentration with the same analytical equipment on the same day. The assay results and statistical evaluation for the assay of the MSB showed %RSD values of 0.89% which is within the acceptable limit of 2.0% as set according to Palestinian Ministry of Health Registration Department criteria.

#### Intermediate Precision (Ruggedness)

Two laboratory analysts carried out the assay of MSB on 12 homogeneous samples of the MSB injectable solution at the 100% level of the final test concentration with two different sets of analytical equipments on two different days. The assay results and statistical evaluation for the assay of the MSB revealed % RSD values of 1.43% which is within the acceptable limit of 2.0%. The results of the assay of the MSB proved that the method is repeatable and rugged enough for day-to-day use.

#### Robustness

Premeditated variations were performed in the experimental conditions of the HPLC method to assess its robustness. The six variations imposed on the chromatographic method are summarized in Table 5. These modifications include different mobile phase flow rates of 0.45, 0.50, and 0.55 ml/min and three different column temperatures in the range 22–28°C. Different NH_4_AC solution concentrations in the mobile phase (190mM, 200mM, and 210mM) and different ACN percentages in the mobile phase (78%, 80%, and 82%) were also investigated. Three column batches filled with the same prescribed stationary phases were studied. Finally, three different pH values of the mobile phase at 5.5, 5.7, and 5.9 were tested. The % RSD values showed no significant change in the final assay results of the MSB using the six variations, and the assay results of MSB showed results between 98.7% and 101.3% with a % RSD of less than 0.84% for the combined results, which are within the acceptable limit (98.0 to 102.0% assay results and %RSD ≤2.0%) (Table 5).

### Applicability of the Method to Marketed Products

It is evident from the results obtained that the validated method gave satisfactory results with respect to the analysis of MSB. The validated method is applied to two different commercially available products manufactured by two different pharmaceutical companies (Vitacare K_3_^®^ injectable solution and Vita-bal K_3_ injectable solution) as shown in Table 6.

This acceptable value indicated the applicability of the proposed method for the routine quality control of the MSB injectable solution.

## Experimental

### Materials and Reagents

The reference standard for MSB 98% (Lot no: 048K1372) was purchased from Sigma-Aldrich (Germany). Ammonium acetate extra pure, glacial acetic acid, HPLC grade acetonitrile (ACN) and methanol (MeOH) solvents, hydrochloric acid fuming (37%), sodium hydroxide pellets, and hydrogen peroxide (30%), were purchased from Merck (Germany). Highly purified water was prepared by using a Millipore Milli-Q Plus water purification system. Vitacare K_3_^®^ injectable solution samples (each milliliter contains 10 mg MSB), MSB active ingredient, excipients (includes water for injection, citric acid, sodium disulfite, and benzyl alcohol) were kindly supplied by Pharmacare pharmaceutical company, Palestine. Vita-bal K_3_ injectable solution (each milliliter contains 10 mg MSB) was purchased from a local pharmacy. The Octadecyl Silane C18 chemically bonded column (250 mm × 4.6 mm i.d., 5 μm particles) was purchased from ACE, United Kingdom.

### Equipment

The HPLC system consisted of LaChrom (Merck-Hitachi) equipped with a model L-7100 pump, L-7200 autosampler, L-7300 column oven, DAD L-7450 photodiode array (PDA) detector, and D-7000 software HSM version 3.1 (Merck Hitachi, England). A double beam ultraviolet-visible spectrometer (PG Instruments, United Kingdom) was used.

A UV-Chamber (Model CM-10) Spectoline fluorescence analysis cabinet was used at 254 nm.

### Chromatographic Conditions

The HPLC experimental conditions were optimized on a ZIC^®^-HILIC column (250 mm ×4.6 mm, 5μm) protected with a ZIC^®^-HILIC guard column (20mm× 2.1mm, 5μm) that was purchased from Merck, Germany.

200mM ammonium acetate solution was prepared by dissolving 3.08 g of NH_4_AC in purified water and diluted up to 200 ml with the same solvent.

The optimum mobile phase was prepared by mixing 200 mM NH_4_AC solution and acetonitrile (ACN) (20:80; v/v), shaken well and left till the temperature of the mobile phase reached room temperature. Then the pH was adjusted to 5.7 with glacial acetic acid. The mobile phase was filtered by using a 0.45 μm microporous filter and was degassed by sonication prior to use. A wavelength of 261 nm was chosen. The flow rate used was 0.5 ml/minute as recommended by the column manufacturer. The injection volume was 20 μl and the temperature of the column was 25°C. The total run time was about 9.0 minutes.

### Preparation of Standard Solution

The standard solution was prepared by dissolving 30 mg of the MSB reference standard in 80 ml of 80% ACN and diluting it up to 100 ml with the same solvent. 5 ml of this solution was diluted up to 50 ml with the mobile phase. This solution was filtered using a 0.45 μm membrane filter before analysis. The obtained final solution contained 30μg/ml MSB. This solution was directly protected from light.

### Preparation of Sample Solution

Three ml of MSB injectable solution was transferred to a 100 ml volumetric flask containing 80 ml of 80% ACN, shaken by mechanical means for 5 minutes, sonicated for 2 minutes, and then diluted up to 100 ml with the same solvent. Using the volumetric pipette, 5 ml of this solution was transferred to the 50 ml volumetric flask and completed to the volume using the mobile phase. This solution was filtered using a 0.45 μm membrane filter before analysis. The obtained final solution contained 30 μg/ml MSB. This solution was directly protected from light.

### Forced Degradation Study

ICH prescribed stress conditions such as acidic, basic, oxidative, thermal, and photolytic stresses, which were carried out.

### Standard Drug Stock Solutions

The forced degradation study was conducted on solutions that were prepared by transferring 30 mg of the MSB reference standard into five different 100 ml volumetric flasks. Then 70 ml of 80% ACN was added to each flask and shaken by mechanical means for 5 minutes, and sonicated for 2 minutes until completely dissolved. These stock solutions were kept at room temperature, protected from light, and used for forced degradation studies.

### Acid Hydrolysis

Ten ml of 1.0 N HCl was added into one of the flasks containing the MSB stock solution and kept at room temperature for 60 minutes in a dark place and then diluted to 100 ml with 80% ACN. Five ml of this solution was transferred into a 50 ml volumetric flask, neutralized with 0.1 N NaOH, and completed to volume using the mobile phase. This solution was filtered using a 0.45 μm membrane filter before analysis. The obtained final solution contained 30 μg/ml MSB.

### Base Hydrolysis

Ten ml of 1.0 N NaOH was added into one of the flasks containing the MSB stock solution and kept at room temperature for 60 minutes in a dark place and then diluted to 100 ml with 80% ACN. Five ml of this solution was transferred into a 50 ml volumetric flask, neutralized with 0.1 N HCl, and completed to volume using the mobile phase. This solution was filtered using a 0.45 μm membrane filter before analysis. The obtained final solution contained 30 μg/ml MSB.

### Oxidative Hydrolysis

Ten ml of 10% H_2_O_2_ was added into one of the flasks containing the menadione sodium bisulfite stock solution and kept at room temperature for 24 hours in a dark place and then diluted to 100 ml with 80% ACN. Five ml of this solution was transferred into a 50 ml volumetric flask and completed to volume using the mobile phase. This solution was filtered using a 0.45 μm membrane filter before analysis. The obtained final solution contained 30 μg/ml MSB.

### Thermal Degradation

One of the flasks containing the MSB stock solution was studied for its thermal degradation by keeping it at 70°C in a water bath protected from light for 96 hours and then diluted to 100 ml with 80% ACN. Five ml of this solution was transferred into a 50 ml volumetric flask and completed to volume using the mobile phase. This solution was filtered using a 0.45 μm membrane filter before analysis. The obtained final solution contained 30μg/ml MSB.

### Photo Degradation

One of the flasks containing the MSB stock solution was studied separately for its photo degradation by exposing it to UV light at 254 nm in a UV-Chamber for 36 hours and then diluted to 100 ml with 80% ACN. Five ml of this solution was transferred into a 50 ml volumetric flask and completed to volume using the mobile phase. This solution was filtered using a 0.45 μm membrane filter before analysis. The obtained final solution contained 30 μg/ml MSB.

### Forced Degradation Study on the MSB Injectable Solution

The sample stock solutions were prepared by separately transferring 3 ml of the MSB injectable solution (containing 10 mg MSB per ml) into a series of five different 100 ml volumetric flasks. The very same procedure adopted for the standard solutions was used in the MSB injectable solution. The obtained final solution contained 30 μg/ml MSB.

### Conclusion

The validated HPLC method developed for the quantitative quality control determination of MSB in the MSB injectable solution was evaluated for system suitability, specificity, sensitivity, linearity, range, accuracy (recovery), precision (repeatability and intermediate precision), and robustness. All the validation results were within the allowed specifications of the ICH/USP guidelines. The developed method proved to be rapid, accurate, and stability-indicating for the determination of MSB in the MSB injectable solution in the presence of excipients, preservatives, and the degradation products. The assay showed complete separation of MSB from its degradation products and from the placebo. As a result, the proposed HPLC method could be adopted for the quantitative quality control and routine analysis of the MSB injectable solution.

## Figures and Tables

**Tab. 1:** Summary of the accepted system suitability requirements

Parameter	MSB	Accepted limit*
% RSD	0.93	≤2.0%
Tailing factor (T_f_)	1.24	≤2.0
Number of theoretical plates (N)	6346	≥3000
Capacity factor (k′)	3.8	≥1.0
Resolution(R_s_)	3.17	≥2.0

* Set according to Palestinian Ministry of Health Registration Department criteria.

**Tab. 2:** Summary of the forced degradation of MSB standard and MSB injectable solution

Name	Stress condition	Degradation %	Purity index*
**MSB standard**	Acidic/0.10 N HCl/60 min at RT	7.87	1.0000
	Alkaline/0.10 N NaOH/60min at RT	59.04	0.9988
	Oxidative/1.0% H_2_O_2_/24 hours at RT	6.67	0.9999
	Thermal/70 °C/96 hours	42.28	0.9991
	Light/ UV-254nm/36 hours	6.81	1.0000

**MSB sample**	Acidic/0.10 N HCl/60 min at RT	7.63	0.9999
	Alkaline/0.10 N NaOH/60min at RT	58.72	0.9986
	Oxidative/1.0% H_2_O_2_/24 hours at RT	6.58	0.9999
	Thermal/70 °C/96 hours	41.84	0.9993
	Light/ UV-254nm/36 hours	6.74	1.0000

* The accepted criteria is > 0.990 that set according to Palestinian Ministry of Health Registration Department criteria. The purity index is a measure of spectral heterogeneity of a peak.

**Tab. 3:** Regression statistics

Active ingredient	Linearity range (μg/ml)	(R^2^)	Linearity equation*	Y-intercept
**MSB**	15–45	0.9996	Y = 279783X + 41236	0.49%

* Y is the dependent variable and X is the independent variable.

**Tab. 4:** Average recoveries, % RSD values at five concentration levels of spiking of MSB

Active ingredient	Amount added (level %)	Average recovery (%)*(n=3)*	RSD (%)*(n=3)*
**MSB**	15 μg/ml (50%)	98.8	0.74
	22.5 μg/ml (75%)	99.1	0.86
	30 μg/ml (100%)	99.4	0.68
	37.5 μg/ml (125%)	99.8	1.13
	45 μg/ml (150%)	98.3	0.83

**Tab. 5:** Robustness testing of the MSB active ingredient

Active ingredient	Parameter	Average assay% *(n=3)*	Average RT (min) *(n=3)*
**MSB**	0.45 ml/min flow	98.9	8.038
	0.50 ml/min flow	99.7	7.206
	0.55 ml/min flow	99.3	6.538
	NH_4_AC: ACN (22:78;v/v)	100.3	6.924
	NH_4_AC: CAN (20:80;v/v)	100.2	7.213
	NH_4_AC: ACN(18:82;v/v)	100.7	7.624
	Temperature (°C)	99.6	7.174
	Buffer Conc.	98.7	7.208
	Column batches	101.3	7.204
	Mobile phase pH	99.1	7.213

**Tab. 6:** Result of market products

Product Name	Labeled claim (mg/ml)	MSB (mg/ml)	Assay%
Vitacare K_3_^®^ injectable solution	10 mg/ml	10.17	101.7%
Vita-bal K_3_ injectable solution	10 mg/ml	9.84	98.4%

## References

[1] Allen DG, Pringle JK, Smith DA, Pasloske K (1998). Handbook of veterinary drugs.

[2] Chiba K, Ishihashi Y, Hayakawa T (1985). Determination of vitamin K3 in premixtures by high-performance liquid chromatography. Shiryo Kenkyu Hokoku (Tokyo Hishiryo Kensasho).

[3] Liu Z, Li T, Li J, Wang E (1997). Detection of menadione sodium bisulfite (vitamin K3) by reversed-phase high performance liquid chromatography with series dual-electrode amperometric detector. Anal Chim Acta.

[4] Billedeau SM (1989). Fluorimetric determination of vitamin K3 (menadione sodium bisulfite) in synthetic animal feed by high-performance liquid chromatography using a post-column zinc reducer. J Chromatogr A.

[5] Zhao C, Li Y, Du H, Xu H, Jiao K (2010). Analysis of vitamin K3 by a fluorescent spectroelectrochemistry method. Chem Res Chin Univ.

[6] Kondo Masao, Wakamatu Mie, Hanai Masahiro (2002). Study on electrochemical determination of vitamin K. Aichi-ken Sangyo Gijutsu Kenkyusho Kenkyu Hokoku.

[7] Ghanem M, Abu-Lafi S, Karaman R, Hallak H (2012). Validated HPLC method to simultaneously determine amprolium hydrochloride, sulfaquinoxaline sodium and vitamin K3 in A.S.K powder on ZIC-HILIC column. Pharm Anal Acta.

[8] British Pharmacopoeia (2005).

[9] United States Pharmacopeia (2011). United State Pharmacopeial Convention, Inc. National formulary, Menadione Monograph.

[10] United States Pharmacopeia (2011). United State Pharmcopeial Convention Inc. National formulary, Menadione Injection Monograph.

[11] Bogusław B, Sylwia N (2012). Hydrophilic interaction liquid chromatography (HILIC)-a powerful separation technique. Anal Bioanal Chem.

[12] ICH Q2 (R1), International Conference of Harmonization (2005). Validation of Analytical Procedures: Text and Methodology.

[13] United States Pharmacopeia (2011). United State Pharmacopeial Convention Inc. National formulary, Validation of compendial procedures.

